# Supplementary material for: Malaria parasites differentially sense environmental elasticity during transmission
Source: EMBO Mol Med. 2021 Mar 5;13(4):e13933. doi: 10.15252/emmm.202113933 (PMC8033522; doi:10.15252/emmm.202113933)
Supplement: Supplementary file 1 — Expanded View Figures PDF [file EMMM-13-e13933-s004.pdf]

Expanded View Figures

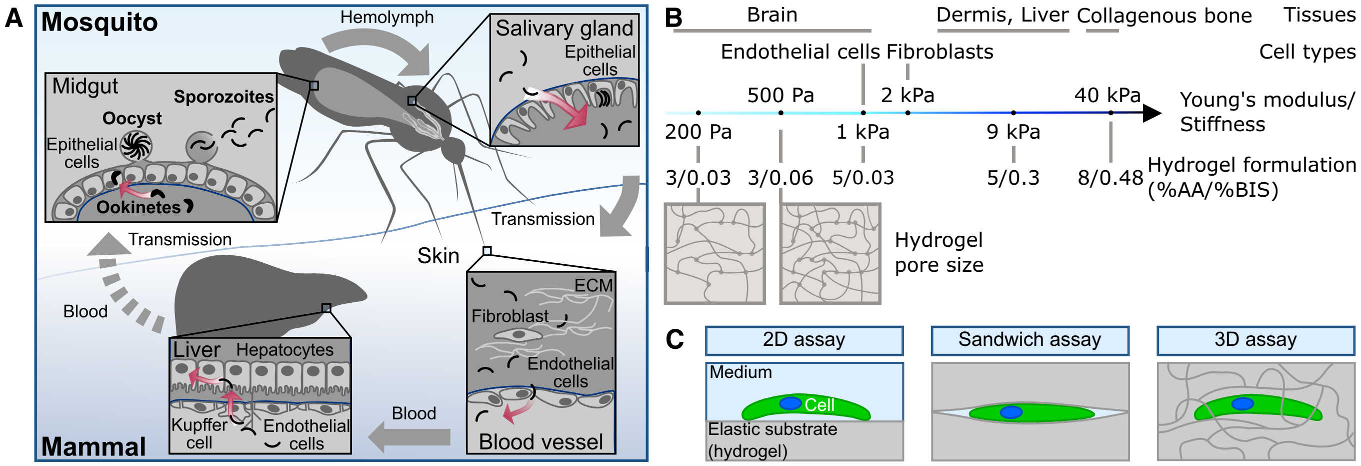

Figure EV1. *Plasmodium* parasites need to traverse and invade different host cells and tissues to progress through the life cycle.

A Ookinetes develop in the midgut and traverse the midgut epithelium to transform into an oocyst at the basement membrane. After rupture of the oocyst, sporozoites are released into the hemolymph. They invade the mosquito salivary glands and move fast upon injection into the dermis via the mosquito bite. Sporozoites enter blood vessels, are transported with the blood circulation, attach to endothelial cells and enter the liver tissue until they finally invade a hepatocyte for transformation and proliferation. Barriers faced by the parasites are depicted in blue. Red arrows indicate active migration across barriers.

B Polyacrylamide hydrogels can be fabricated to mimic the stiffness of different tissues (Engler *et al*, 2006) and cell types (Grady *et al*, 2016) by adjusting the monomer to crosslinker concentration (Tse & Engler, 2010). A higher amount of crosslinker leads to smaller pores and higher elastic moduli of the gels.

C Experimental setups used in this study to investigate the effect of substrate elasticity, confinement and pores size on ookinete and sporozoite motility.

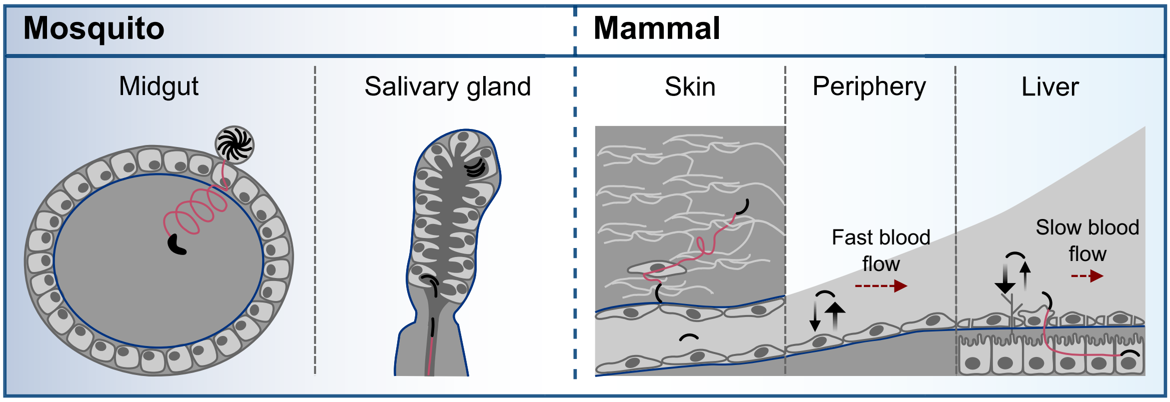

Figure EV2. Scheme illustrating different modes of motility throughout the *Plasmodium* life cycle.

Robust ookinete motility on soft substrates might allow for active dissemination within the blood bolus in the mosquito midgut with a change in migration mode upon contact of and passing through the epithelial cells. During an infectious bite, sporozoites need to pass through the narrow salivary duct to be transmitted into the mammalian skin. Once in the skin, sporozoites migrate and squeeze through pores to find and invade a blood vessel. With the blood flow (red arrow), sporozoites are transported through the body. The elasticity of endothelial cells does not favor adhesion (black arrows) so that sporozoites only arrest upon reaching the liver sinusoids where they escape the blood stream and infect hepatocytes (red line).
