# Supplementary material for: Malaria parasites differentially sense environmental elasticity during transmission
Source: EMBO Mol Med. 2021 Mar 5;13(4):e13933. doi: 10.15252/emmm.202113933 (PMC8033522; doi:10.15252/emmm.202113933)
Supplement: Supplementary file 2 — Movie EV1 [file EMMM-13-e13933-s007.zip › Movie_EV1/Movie_EV1.docx]

Movie showing ookinetes sandwiched between two PA hydrogels. Differential interference contrast (DIC) images were taken every 20 s. Scale bar, 20 µm.
