# Supplementary material for: Malaria parasites differentially sense environmental elasticity during transmission
Source: EMBO Mol Med. 2021 Mar 5;13(4):e13933. doi: 10.15252/emmm.202113933 (PMC8033522; doi:10.15252/emmm.202113933)
Supplement: Supplementary file 3 — Movie EV2 [file EMMM-13-e13933-s006.zip › Movie_EV2.docx]

Movie showing sporozoites moving through a PA hydrogel. Images were recorded using confocal spinning-disk microscopy.
